# Cabraleahy­droxy­lactone from the leaves of *Aglaia exima*

**DOI:** 10.1107/S1600536810035208

**Published:** 2010-09-11

**Authors:** Xe Min Loong, Khalit Mohamad, Khalijah Awang, A. Hamid A. Hadi, Seik Weng Ng

**Affiliations:** a Department of Chemistry, University of Malaya, 50603 Kuala Lumpur, Malaysia; b Department of Pharmacy, University of Malaya, 50603 Kuala Lumpur, Malaysia

## Abstract

Cabraleahy­droxy­lactone, C_27_H_44_O_3_, isolated from the leaves of *Aglaia exima*, has three six-membered rings fused together that adopt chair conformations. Its two five-membered rings are enveloped shaped. The hy­droxy group is in an axial position. It is a hydrogen-bond donor to the carbonyl O atom of an adjacent mol­ecule; the O—H⋯O inter­actions lead to the formation of a helical chain that runs along the *b* axis. There are two independent mol­ecules in the asymmetric unit.

## Related literature

For the isolation and spectroscopic characterization of cabraleahy­droxy­lactone from other *Alglaia* species, see: Su *et al.* (2006 ▶); Yang *et al.* (2008 ▶). For another compound from *Aglaia exima*, see: Awang *et al.* (2010 ▶).
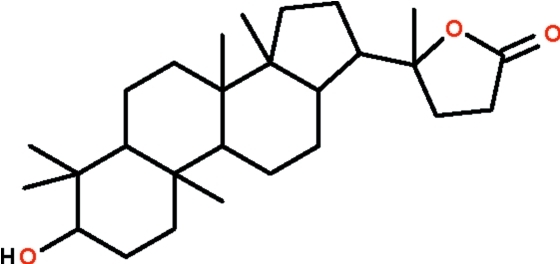


## Experimental

### 

#### Crystal data

C_27_H_44_O_3_
*M* *_r_* = 416.62
Orthorhombic, 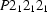

*a* = 7.2077 (5) Å
*b* = 20.8363 (15) Å
*c* = 30.464 (2) Å
*V* = 4575.2 (6) Å^3^
*Z* = 8
Mo *K*α radiation
μ = 0.08 mm^−1^
*T* = 100 K
0.30 × 0.05 × 0.05 mm

#### Data collection

Bruker SMART APEX diffractometer
36097 measured reflections
4573 independent reflections
3803 reflections with *I* > 2σ(*I*)
*R*_int_ = 0.098

#### Refinement

*R*[*F* ^2^ > 2σ(*F* ^2^)] = 0.047
*wR*(*F* ^2^) = 0.121
*S* = 1.07
4573 reflections
555 parameters
H-atom parameters constrained
Δρ_max_ = 0.26 e Å^−3^
Δρ_min_ = −0.25 e Å^−3^

### 

Data collection: *APEX2* (Bruker, 2009 ▶); cell refinement: *SAINT* (Bruker, 2009 ▶); data reduction: *SAINT*; program(s) used to solve structure: *SHELXS97* (Sheldrick, 2008 ▶); program(s) used to refine structure: *SHELXL97* (Sheldrick, 2008 ▶); molecular graphics: *X-SEED* (Barbour, 2001 ▶); software used to prepare material for publication: *publCIF* (Westrip, 2010 ▶).

## Supplementary Material

Crystal structure: contains datablocks global, I. DOI: 10.1107/S1600536810035208/bt5334sup1.cif

Structure factors: contains datablocks I. DOI: 10.1107/S1600536810035208/bt5334Isup2.hkl

Additional supplementary materials:  crystallographic information; 3D view; checkCIF report

## Figures and Tables

**Table 1:** Hydrogen-bond geometry (Å, °)

*D*—H⋯*A*	*D*—H	H⋯*A*	*D*⋯*A*	*D*—H⋯*A*
O1—H1⋯O4^i^	0.84	2.02	2.836 (4)	163
O4—H4⋯O2	0.84	2.03	2.858 (4)	169

Symmetry code: (i) 
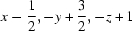
.

**Fig. 1.**
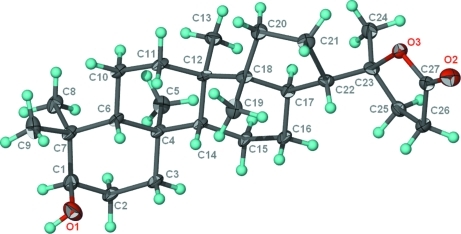
Anisotropic displacement ellipsoid plot (Barbour, 2001) of one of the two independent molecules of C27H44O3 at the 70% probability level; hydrogen atoms are drawn as spheres of arbitrary radius.

**Fig. 2.**
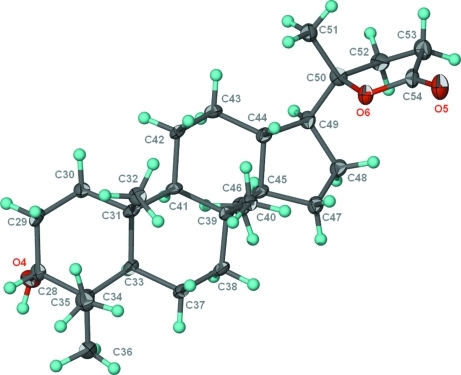
Anisotropic displacement ellipsoid plot (Barbour, 2001) of the second independent molecule of C27H44O3 at the 70% probability level; hydrogen atoms are drawn as spheres of arbitrary radius.

## supplementary crystallographic information

### Comment

The *Aglaia* genus consists of some 130 species of trees and shrubs distributed mainly in the tropical rain forests of Southeast Asia. Cabraleahydroxylactone (Scheme I) has been isolated from *Aglaia crassinervia* (Su *et al.*, 2006) and *Aglaia perviridis* (Yang *et al.*, 2008) as well as from other plants, and the structure and absolute configuration have been established from spectroscopic measurements. The structural assignment is confirmed by the present crystal structure analysis of the compound isolated from *Aglaia exima*. There are two independent cabraleahydroxylactone molecules in the asymmetric unit. Each molecule has three six-membered rings fused together that adopt chair conformations. The two five-membered rings are enveloped-shaped (Figs. 1 and 2). The hydroxy unit is axial; this unit is hydrogen-bond donor to the double-bond oxygen atom of an adjacent molecule. The O–H···O interactions lead to the formation of a chain that runs along the longest axis of the orthorhombic unit cell.

### Experimental

The leaves of *Algaia exima* were collected from Kampung Kepayang, Pahang, Malaysia. The leaves (1 kg) were soaked in *n*-hexane for 4 days. The solution was decanted and then evaporated to leave a residue of 25 g; a 15 g portion was subjected to column chromatography over silica gel at a gradient mixture of *n*-hexane and ethyl acetate. Of the 123 fractions, fractions 94–100 were separated by column chromatography (7:3 *n*-hexane:ethyl acetate) to give a colorless solid. Single crystals of cabraleahydroxylactone were obtained by recrystallization from ethyl acetate.

### Refinement

Carbon-bound H-atoms were placed in calculated positions [C—H 0.95 to 1.00 Å, *U*(H) 1.2 to 1.5*U*(C)] and were included in the refinement in the riding model approximation. The torsion angles of the methyl groups were refined. The hydroxy H-atoms were similarly placed [O–H 0.84 Å] and their displacement parameters were set to  1.5*U*(O).

3940 Friedel pairs were merged.

### Crystal data

**Table:**

C_27_H_44_O_3_	*F*(000) = 1840
*M**_r_* = 416.62	*D*_x_ = 1.210 Mg m^−^^3^
Orthorhombic, *P*2_1_2_1_2_1_	Mo *K*α radiation, λ = 0.71073 Å
Hall symbol: P 2ac 2ab	Cell parameters from 4625 reflections
*a* = 7.2077 (5) Å	θ = 2.2–24.2°
*b* = 20.8363 (15) Å	µ = 0.08 mm^−^^1^
*c* = 30.464 (2) Å	*T* = 100 K
*V* = 4575.2 (6) Å^3^	Prism, colorless
*Z* = 8	0.30 × 0.05 × 0.05 mm

### Data collection

**Table:**

Bruker SMART APEX diffractometer	3803 reflections with *I* > 2σ(*I*)
Radiation source: fine-focus sealed tube	*R*_int_ = 0.098
graphite	θ_max_ = 25.0°, θ_min_ = 1.2°
ω scans	*h* = −8→8
36097 measured reflections	*k* = −24→23
4573 independent reflections	*l* = −36→36

### Refinement

**Table:**

Refinement on *F*^2^	Primary atom site location: structure-invariant direct methods
Least-squares matrix: full	Secondary atom site location: difference Fourier map
*R*[*F*^2^ > 2σ(*F*^2^)] = 0.047	Hydrogen site location: inferred from neighbouring sites
*wR*(*F*^2^) = 0.121	H-atom parameters constrained
*S* = 1.07	*w* = 1/[σ^2^(*F*_o_^2^) + (0.0442*P*)^2^ + 4.0407*P*] where *P* = (*F*_o_^2^ + 2*F*_c_^2^)/3
4573 reflections	(Δ/σ)_max_ = 0.001
555 parameters	Δρ_max_ = 0.26 e Å^−^^3^
0 restraints	Δρ_min_ = −0.25 e Å^−^^3^

### Fractional atomic coordinates and isotropic or equivalent isotropic displacement parameters (Å^2^)

**Table:**

	*x*	*y*	*z*	*U*_iso_*/*U*_eq_	
O1	0.2796 (5)	0.58659 (13)	0.38176 (9)	0.0281 (7)	
H1	0.2911	0.5471	0.3770	0.042*	
O2	0.4571 (4)	1.09025 (13)	0.60025 (8)	0.0251 (7)	
O3	0.3075 (4)	1.06676 (12)	0.53818 (8)	0.0171 (6)	
O4	0.7658 (4)	1.04183 (13)	0.64896 (8)	0.0204 (6)	
H4	0.6726	1.0594	0.6376	0.031*	
O5	0.9808 (4)	0.79695 (14)	1.01455 (9)	0.0278 (7)	
O6	0.9900 (4)	0.79834 (12)	0.94154 (8)	0.0176 (6)	
C1	0.2089 (6)	0.61757 (19)	0.34291 (12)	0.0191 (9)	
H1A	0.1912	0.5842	0.3197	0.023*	
C2	0.3559 (6)	0.66442 (19)	0.32737 (13)	0.0208 (9)	
H2A	0.3199	0.6817	0.2983	0.025*	
H2B	0.4753	0.6415	0.3239	0.025*	
C3	0.3817 (5)	0.72036 (19)	0.35968 (13)	0.0178 (8)	
H3A	0.4273	0.7033	0.3880	0.021*	
H3B	0.4773	0.7499	0.3480	0.021*	
C4	0.2018 (5)	0.75810 (18)	0.36769 (12)	0.0143 (8)	
C5	0.1559 (6)	0.79549 (19)	0.32509 (11)	0.0185 (9)	
H5A	0.2078	0.7727	0.2998	0.028*	
H5B	0.2098	0.8386	0.3267	0.028*	
H5C	0.0210	0.7988	0.3218	0.028*	
C6	0.0509 (5)	0.70880 (18)	0.38243 (12)	0.0142 (8)	
H6	0.0993	0.6905	0.4105	0.017*	
C7	0.0202 (5)	0.64947 (18)	0.35207 (12)	0.0170 (8)	
C8	−0.0750 (6)	0.6656 (2)	0.30844 (12)	0.0211 (9)	
H8A	0.0071	0.6928	0.2907	0.032*	
H8B	−0.1911	0.6886	0.3142	0.032*	
H8C	−0.1018	0.6259	0.2925	0.032*	
C9	−0.1035 (6)	0.59982 (19)	0.37536 (13)	0.0246 (10)	
H9A	−0.0986	0.5590	0.3594	0.037*	
H9B	−0.2317	0.6154	0.3761	0.037*	
H9C	−0.0590	0.5934	0.4054	0.037*	
C10	−0.1287 (5)	0.74284 (18)	0.39526 (12)	0.0160 (8)	
H10A	−0.2243	0.7106	0.4030	0.019*	
H10B	−0.1752	0.7682	0.3701	0.019*	
C11	−0.0943 (5)	0.78739 (19)	0.43454 (12)	0.0154 (8)	
H11A	−0.0567	0.7611	0.4601	0.019*	
H11B	−0.2118	0.8093	0.4422	0.019*	
C12	0.0556 (5)	0.83837 (18)	0.42601 (12)	0.0129 (8)	
C13	−0.0256 (6)	0.88744 (18)	0.39315 (12)	0.0187 (9)	
H13A	−0.1171	0.9145	0.4081	0.028*	
H13B	−0.0857	0.8645	0.3689	0.028*	
H13C	0.0744	0.9144	0.3816	0.028*	
C14	0.2335 (5)	0.80400 (18)	0.40804 (11)	0.0122 (8)	
H14	0.2734	0.7749	0.4324	0.015*	
C15	0.3967 (5)	0.85132 (19)	0.40226 (13)	0.0173 (8)	
H15A	0.5085	0.8272	0.3931	0.021*	
H15B	0.3660	0.8822	0.3787	0.021*	
C16	0.4400 (5)	0.88847 (19)	0.44474 (13)	0.0175 (8)	
H16A	0.4863	0.8584	0.4674	0.021*	
H16B	0.5381	0.9207	0.4390	0.021*	
C17	0.2675 (5)	0.92170 (17)	0.46118 (12)	0.0139 (8)	
H17	0.2242	0.9505	0.4370	0.017*	
C18	0.1089 (5)	0.87326 (18)	0.46999 (12)	0.0138 (8)	
C19	0.1657 (6)	0.82524 (19)	0.50616 (12)	0.0199 (9)	
H19A	0.2378	0.8476	0.5287	0.030*	
H19B	0.2411	0.7910	0.4933	0.030*	
H19C	0.0541	0.8067	0.5194	0.030*	
C20	−0.0365 (6)	0.91833 (18)	0.49069 (12)	0.0188 (9)	
H20A	−0.0975	0.9449	0.4680	0.023*	
H20B	−0.1324	0.8935	0.5066	0.023*	
C21	0.0764 (5)	0.96028 (19)	0.52249 (13)	0.0186 (8)	
H21A	0.0775	0.9410	0.5522	0.022*	
H21B	0.0221	1.0038	0.5244	0.022*	
C22	0.2785 (5)	0.96349 (17)	0.50328 (12)	0.0149 (8)	
H22	0.3646	0.9421	0.5245	0.018*	
C23	0.3445 (5)	1.03260 (18)	0.49629 (12)	0.0155 (8)	
C24	0.2401 (6)	1.06900 (18)	0.46072 (12)	0.0192 (9)	
H24A	0.2721	1.1147	0.4622	0.029*	
H24B	0.1062	1.0638	0.4651	0.029*	
H24C	0.2746	1.0520	0.4319	0.029*	
C25	0.5553 (6)	1.03897 (18)	0.49164 (12)	0.0184 (9)	
H25A	0.6083	1.0014	0.4762	0.022*	
H25B	0.5885	1.0784	0.4753	0.022*	
C26	0.6246 (6)	1.0423 (2)	0.53885 (13)	0.0216 (9)	
H26A	0.6581	0.9992	0.5500	0.026*	
H26B	0.7336	1.0709	0.5414	0.026*	
C27	0.4611 (6)	1.06908 (18)	0.56309 (13)	0.0184 (9)	
C28	0.8607 (6)	1.08736 (18)	0.67638 (12)	0.0162 (8)	
H28	0.8733	1.1285	0.6597	0.019*	
C29	1.0527 (5)	1.06106 (18)	0.68467 (12)	0.0176 (8)	
H29A	1.1296	1.0949	0.6984	0.021*	
H29B	1.1104	1.0497	0.6562	0.021*	
C30	1.0532 (5)	1.00155 (17)	0.71448 (11)	0.0154 (8)	
H30A	1.1831	0.9884	0.7199	0.019*	
H30B	0.9904	0.9658	0.6990	0.019*	
C31	0.9560 (5)	1.01284 (17)	0.75894 (12)	0.0139 (8)	
C32	1.0809 (6)	1.05702 (18)	0.78677 (13)	0.0187 (9)	
H32A	1.1486	1.0864	0.7674	0.028*	
H32B	1.1695	1.0309	0.8034	0.028*	
H32C	1.0041	1.0818	0.8072	0.028*	
C33	0.7601 (5)	1.04067 (17)	0.74876 (12)	0.0140 (8)	
H33	0.6976	1.0065	0.7311	0.017*	
C34	0.7524 (6)	1.10136 (17)	0.71919 (12)	0.0157 (8)	
C35	0.8312 (6)	1.16297 (18)	0.74021 (13)	0.0216 (9)	
H35A	0.7960	1.2002	0.7224	0.032*	
H35B	0.9667	1.1600	0.7417	0.032*	
H35C	0.7808	1.1679	0.7699	0.032*	
C36	0.5499 (6)	1.11633 (19)	0.70719 (13)	0.0202 (9)	
H36A	0.5469	1.1470	0.6828	0.030*	
H36B	0.4866	1.1349	0.7327	0.030*	
H36C	0.4869	1.0767	0.6984	0.030*	
C37	0.6416 (5)	1.04637 (18)	0.79052 (12)	0.0162 (8)	
H37A	0.7045	1.0749	0.8118	0.019*	
H37B	0.5201	1.0657	0.7831	0.019*	
C38	0.6115 (5)	0.98016 (18)	0.81121 (12)	0.0165 (8)	
H38A	0.5358	0.9852	0.8381	0.020*	
H38B	0.5404	0.9531	0.7905	0.020*	
C39	0.7913 (5)	0.94578 (18)	0.82302 (12)	0.0137 (8)	
C40	0.8788 (6)	0.98119 (18)	0.86242 (12)	0.0165 (8)	
H40A	0.8759	1.0276	0.8571	0.025*	
H40B	1.0077	0.9672	0.8661	0.025*	
H40C	0.8085	0.9713	0.8891	0.025*	
C41	0.9237 (5)	0.94567 (17)	0.78171 (12)	0.0134 (8)	
H41	0.8549	0.9200	0.7592	0.016*	
C42	1.1012 (5)	0.90678 (17)	0.79026 (12)	0.0152 (8)	
H42A	1.1759	0.9056	0.7630	0.018*	
H42B	1.1755	0.9288	0.8130	0.018*	
C43	1.0627 (5)	0.83765 (18)	0.80528 (12)	0.0160 (8)	
H43A	1.0020	0.8134	0.7813	0.019*	
H43B	1.1809	0.8160	0.8126	0.019*	
C44	0.9364 (5)	0.83886 (18)	0.84565 (12)	0.0147 (8)	
H44	1.0012	0.8653	0.8683	0.018*	
C45	0.7527 (5)	0.87339 (18)	0.83525 (12)	0.0145 (8)	
C46	0.6461 (6)	0.83813 (19)	0.79815 (13)	0.0198 (9)	
H46A	0.6665	0.7918	0.8008	0.030*	
H46B	0.6913	0.8530	0.7696	0.030*	
H46C	0.5132	0.8473	0.8007	0.030*	
C47	0.6450 (6)	0.86004 (18)	0.87787 (12)	0.0181 (9)	
H47A	0.5112	0.8693	0.8742	0.022*	
H47B	0.6941	0.8860	0.9025	0.022*	
C48	0.6785 (5)	0.78814 (19)	0.88553 (13)	0.0185 (9)	
H48A	0.5870	0.7621	0.8691	0.022*	
H48B	0.6686	0.7776	0.9171	0.022*	
C49	0.8794 (5)	0.77520 (18)	0.86838 (12)	0.0162 (8)	
H49	0.8726	0.7409	0.8454	0.019*	
C50	1.0103 (6)	0.75229 (18)	0.90459 (12)	0.0168 (8)	
C51	1.2139 (5)	0.75294 (19)	0.89235 (13)	0.0187 (9)	
H51A	1.2529	0.7971	0.8862	0.028*	
H51B	1.2333	0.7264	0.8662	0.028*	
H51C	1.2874	0.7358	0.9167	0.028*	
C52	0.9517 (6)	0.68784 (18)	0.92501 (12)	0.0197 (9)	
H52A	0.8179	0.6797	0.9203	0.024*	
H52B	1.0239	0.6519	0.9124	0.024*	
C53	0.9947 (6)	0.69623 (19)	0.97360 (13)	0.0239 (9)	
H53A	0.9010	0.6743	0.9920	0.029*	
H53B	1.1191	0.6791	0.9808	0.029*	
C54	0.9876 (5)	0.76753 (19)	0.98025 (12)	0.0190 (9)	

### Atomic displacement parameters (Å^2^)

**Table:**

	*U*^11^	*U*^22^	*U*^33^	*U*^12^	*U*^13^	*U*^23^
O1	0.0374 (19)	0.0199 (15)	0.0270 (16)	0.0070 (15)	−0.0114 (14)	−0.0033 (13)
O2	0.0267 (17)	0.0304 (16)	0.0183 (14)	0.0063 (14)	−0.0016 (13)	−0.0066 (12)
O3	0.0152 (14)	0.0202 (14)	0.0161 (13)	0.0014 (12)	−0.0013 (11)	−0.0061 (11)
O4	0.0211 (16)	0.0233 (15)	0.0169 (14)	0.0035 (13)	−0.0060 (12)	−0.0032 (11)
O5	0.0351 (19)	0.0317 (16)	0.0164 (15)	−0.0028 (15)	0.0027 (13)	0.0005 (13)
O6	0.0212 (15)	0.0183 (14)	0.0133 (13)	−0.0027 (12)	0.0012 (12)	−0.0005 (11)
C1	0.019 (2)	0.022 (2)	0.016 (2)	−0.0013 (18)	−0.0033 (17)	−0.0042 (16)
C2	0.0093 (19)	0.028 (2)	0.025 (2)	0.0007 (18)	0.0018 (17)	−0.0101 (18)
C3	0.0091 (19)	0.024 (2)	0.020 (2)	0.0003 (17)	−0.0013 (16)	−0.0040 (17)
C4	0.0081 (18)	0.020 (2)	0.0145 (18)	−0.0009 (16)	0.0009 (15)	−0.0016 (15)
C5	0.022 (2)	0.022 (2)	0.0112 (18)	−0.0013 (18)	0.0020 (17)	−0.0011 (16)
C6	0.0093 (18)	0.0196 (19)	0.0136 (18)	−0.0005 (17)	−0.0027 (15)	0.0004 (15)
C7	0.0107 (19)	0.021 (2)	0.0191 (19)	−0.0022 (17)	0.0003 (16)	−0.0002 (16)
C8	0.016 (2)	0.028 (2)	0.019 (2)	−0.0044 (19)	0.0032 (17)	−0.0059 (17)
C9	0.032 (3)	0.020 (2)	0.022 (2)	−0.0082 (19)	0.0024 (19)	−0.0029 (17)
C10	0.0109 (19)	0.021 (2)	0.0162 (19)	−0.0009 (17)	−0.0009 (16)	0.0005 (16)
C11	0.0061 (19)	0.024 (2)	0.0160 (19)	0.0005 (17)	0.0038 (15)	−0.0009 (16)
C12	0.0067 (18)	0.0164 (19)	0.0158 (18)	0.0016 (16)	−0.0002 (15)	0.0010 (15)
C13	0.019 (2)	0.0172 (19)	0.020 (2)	0.0053 (17)	−0.0036 (17)	0.0006 (16)
C14	0.0068 (18)	0.0188 (19)	0.0110 (17)	−0.0002 (16)	0.0012 (15)	−0.0003 (15)
C15	0.0082 (19)	0.023 (2)	0.021 (2)	−0.0038 (16)	0.0027 (16)	−0.0060 (17)
C16	0.0085 (19)	0.021 (2)	0.023 (2)	−0.0008 (17)	0.0016 (16)	−0.0057 (17)
C17	0.0129 (19)	0.0165 (19)	0.0124 (17)	−0.0002 (17)	0.0021 (15)	−0.0012 (15)
C18	0.0106 (19)	0.0179 (19)	0.0129 (18)	0.0013 (16)	0.0045 (15)	0.0009 (15)
C19	0.024 (2)	0.023 (2)	0.0124 (19)	−0.0044 (18)	0.0005 (17)	0.0002 (16)
C20	0.014 (2)	0.022 (2)	0.020 (2)	−0.0010 (18)	0.0049 (16)	−0.0044 (16)
C21	0.017 (2)	0.022 (2)	0.0174 (19)	0.0003 (17)	0.0063 (17)	−0.0013 (16)
C22	0.016 (2)	0.0170 (19)	0.0119 (18)	0.0046 (17)	−0.0032 (16)	−0.0011 (15)
C23	0.015 (2)	0.018 (2)	0.0132 (18)	0.0048 (17)	0.0008 (16)	−0.0066 (15)
C24	0.020 (2)	0.018 (2)	0.0198 (19)	−0.0003 (18)	−0.0023 (17)	−0.0001 (16)
C25	0.019 (2)	0.0148 (19)	0.021 (2)	0.0011 (17)	0.0029 (17)	−0.0036 (16)
C26	0.014 (2)	0.021 (2)	0.030 (2)	0.0013 (18)	−0.0030 (18)	−0.0064 (18)
C27	0.018 (2)	0.0152 (19)	0.022 (2)	0.0025 (17)	−0.0041 (17)	−0.0011 (16)
C28	0.018 (2)	0.0168 (19)	0.0137 (18)	−0.0003 (17)	−0.0001 (16)	0.0004 (15)
C29	0.014 (2)	0.023 (2)	0.0167 (19)	−0.0014 (18)	0.0061 (16)	0.0000 (16)
C30	0.0111 (19)	0.020 (2)	0.0155 (18)	0.0000 (17)	−0.0010 (16)	0.0010 (15)
C31	0.0079 (19)	0.0154 (19)	0.0183 (19)	−0.0017 (16)	0.0012 (16)	−0.0023 (15)
C32	0.017 (2)	0.019 (2)	0.020 (2)	−0.0034 (17)	−0.0013 (17)	0.0014 (16)
C33	0.0115 (19)	0.0171 (19)	0.0135 (18)	−0.0004 (17)	0.0019 (16)	−0.0029 (15)
C34	0.015 (2)	0.0149 (19)	0.0168 (19)	0.0009 (17)	0.0024 (17)	0.0005 (15)
C35	0.026 (2)	0.017 (2)	0.021 (2)	0.0003 (19)	−0.0001 (18)	0.0000 (16)
C36	0.018 (2)	0.022 (2)	0.021 (2)	0.0013 (18)	0.0021 (17)	0.0018 (16)
C37	0.0122 (19)	0.020 (2)	0.0161 (19)	0.0028 (17)	0.0027 (16)	−0.0011 (16)
C38	0.0103 (19)	0.022 (2)	0.0169 (19)	0.0025 (17)	0.0030 (16)	−0.0010 (16)
C39	0.0094 (19)	0.019 (2)	0.0123 (18)	0.0012 (16)	0.0004 (15)	−0.0023 (15)
C40	0.017 (2)	0.019 (2)	0.0131 (18)	0.0025 (17)	−0.0004 (16)	0.0002 (15)
C41	0.0076 (18)	0.0174 (19)	0.0152 (18)	0.0019 (16)	−0.0016 (15)	−0.0014 (15)
C42	0.0092 (19)	0.020 (2)	0.0167 (19)	0.0011 (16)	0.0035 (15)	0.0002 (15)
C43	0.0114 (19)	0.0181 (19)	0.0184 (19)	0.0027 (17)	−0.0004 (16)	−0.0008 (16)
C44	0.0105 (19)	0.0189 (19)	0.0149 (18)	−0.0013 (17)	−0.0005 (16)	−0.0007 (15)
C45	0.0075 (18)	0.021 (2)	0.0148 (18)	−0.0001 (17)	−0.0002 (16)	−0.0025 (15)
C46	0.016 (2)	0.020 (2)	0.023 (2)	−0.0026 (18)	−0.0007 (18)	0.0002 (17)
C47	0.013 (2)	0.025 (2)	0.0165 (19)	−0.0029 (17)	0.0002 (17)	0.0028 (16)
C48	0.013 (2)	0.024 (2)	0.0183 (19)	−0.0026 (17)	0.0003 (16)	0.0012 (17)
C49	0.016 (2)	0.0168 (19)	0.0158 (19)	−0.0032 (17)	0.0002 (16)	0.0011 (15)
C50	0.019 (2)	0.0140 (19)	0.0172 (19)	−0.0017 (17)	−0.0017 (17)	−0.0010 (15)
C51	0.019 (2)	0.020 (2)	0.017 (2)	−0.0007 (18)	0.0003 (17)	0.0019 (16)
C52	0.016 (2)	0.018 (2)	0.026 (2)	−0.0023 (17)	0.0002 (18)	0.0035 (16)
C53	0.023 (2)	0.025 (2)	0.024 (2)	−0.0038 (19)	0.0006 (19)	0.0075 (17)
C54	0.016 (2)	0.027 (2)	0.0141 (19)	−0.0037 (18)	0.0009 (16)	0.0053 (17)

### Geometric parameters (Å, °)

**Table:**

O1—C1	1.441 (5)	C25—H25A	0.9900
O1—H1	0.8400	C25—H25B	0.9900
O2—C27	1.216 (4)	C26—C27	1.499 (6)
O3—C27	1.343 (5)	C26—H26A	0.9900
O3—C23	1.485 (4)	C26—H26B	0.9900
O4—C28	1.437 (5)	C28—C29	1.510 (5)
O4—H4	0.8400	C28—C34	1.548 (5)
O5—C54	1.213 (5)	C28—H28	1.0000
O6—C54	1.343 (4)	C29—C30	1.537 (5)
O6—C50	1.486 (4)	C29—H29A	0.9900
C1—C2	1.516 (6)	C29—H29B	0.9900
C1—C7	1.539 (5)	C30—C31	1.543 (5)
C1—H1A	1.0000	C30—H30A	0.9900
C2—C3	1.537 (5)	C30—H30B	0.9900
C2—H2A	0.9900	C31—C32	1.542 (5)
C2—H2B	0.9900	C31—C33	1.557 (5)
C3—C4	1.536 (5)	C31—C41	1.579 (5)
C3—H3A	0.9900	C32—H32A	0.9800
C3—H3B	0.9900	C32—H32B	0.9800
C4—C5	1.549 (5)	C32—H32C	0.9800
C4—C6	1.562 (5)	C33—C37	1.537 (5)
C4—C14	1.574 (5)	C33—C34	1.554 (5)
C5—H5A	0.9800	C33—H33	1.0000
C5—H5B	0.9800	C34—C36	1.537 (6)
C5—H5C	0.9800	C34—C35	1.543 (5)
C6—C10	1.527 (5)	C35—H35A	0.9800
C6—C7	1.560 (5)	C35—H35B	0.9800
C6—H6	1.0000	C35—H35C	0.9800
C7—C8	1.533 (5)	C36—H36A	0.9800
C7—C9	1.539 (5)	C36—H36B	0.9800
C8—H8A	0.9800	C36—H36C	0.9800
C8—H8B	0.9800	C37—C38	1.532 (5)
C8—H8C	0.9800	C37—H37A	0.9900
C9—H9A	0.9800	C37—H37B	0.9900
C9—H9B	0.9800	C38—C39	1.524 (5)
C9—H9C	0.9800	C38—H38A	0.9900
C10—C11	1.535 (5)	C38—H38B	0.9900
C10—H10A	0.9900	C39—C40	1.544 (5)
C10—H10B	0.9900	C39—C41	1.579 (5)
C11—C12	1.537 (5)	C39—C45	1.578 (5)
C11—H11A	0.9900	C40—H40A	0.9800
C11—H11B	0.9900	C40—H40B	0.9800
C12—C13	1.546 (5)	C40—H40C	0.9800
C12—C14	1.567 (5)	C41—C42	1.536 (5)
C12—C18	1.572 (5)	C41—H41	1.0000
C13—H13A	0.9800	C42—C43	1.537 (5)
C13—H13B	0.9800	C42—H42A	0.9900
C13—H13C	0.9800	C42—H42B	0.9900
C14—C15	1.545 (5)	C43—C44	1.530 (5)
C14—H14	1.0000	C43—H43A	0.9900
C15—C16	1.540 (5)	C43—H43B	0.9900
C15—H15A	0.9900	C44—C45	1.540 (5)
C15—H15B	0.9900	C44—C49	1.552 (5)
C16—C17	1.509 (5)	C44—H44	1.0000
C16—H16A	0.9900	C45—C47	1.538 (5)
C16—H16B	0.9900	C45—C46	1.551 (5)
C17—C18	1.548 (5)	C46—H46A	0.9800
C17—C22	1.552 (5)	C46—H46B	0.9800
C17—H17	1.0000	C46—H46C	0.9800
C18—C20	1.542 (5)	C47—C48	1.535 (5)
C18—C19	1.544 (5)	C47—H47A	0.9900
C19—H19A	0.9800	C47—H47B	0.9900
C19—H19B	0.9800	C48—C49	1.563 (5)
C19—H19C	0.9800	C48—H48A	0.9900
C20—C21	1.538 (5)	C48—H48B	0.9900
C20—H20A	0.9900	C49—C50	1.528 (5)
C20—H20B	0.9900	C49—H49	1.0000
C21—C22	1.572 (5)	C50—C51	1.514 (6)
C21—H21A	0.9900	C50—C52	1.539 (5)
C21—H21B	0.9900	C51—H51A	0.9800
C22—C23	1.531 (5)	C51—H51B	0.9800
C22—H22	1.0000	C51—H51C	0.9800
C23—C24	1.522 (5)	C52—C53	1.523 (5)
C23—C25	1.532 (6)	C52—H52A	0.9900
C24—H24A	0.9800	C52—H52B	0.9900
C24—H24B	0.9800	C53—C54	1.500 (6)
C24—H24C	0.9800	C53—H53A	0.9900
C25—C26	1.524 (5)	C53—H53B	0.9900
			
C1—O1—H1	109.5	O2—C27—C26	127.8 (4)
C27—O3—C23	110.8 (3)	O3—C27—C26	110.9 (3)
C28—O4—H4	109.5	O4—C28—C29	107.1 (3)
C54—O6—C50	111.0 (3)	O4—C28—C34	112.0 (3)
O1—C1—C2	107.3 (3)	C29—C28—C34	112.9 (3)
O1—C1—C7	110.9 (3)	O4—C28—H28	108.2
C2—C1—C7	113.3 (3)	C29—C28—H28	108.2
O1—C1—H1A	108.4	C34—C28—H28	108.2
C2—C1—H1A	108.4	C28—C29—C30	113.2 (3)
C7—C1—H1A	108.4	C28—C29—H29A	108.9
C1—C2—C3	111.9 (3)	C30—C29—H29A	108.9
C1—C2—H2A	109.2	C28—C29—H29B	108.9
C3—C2—H2A	109.2	C30—C29—H29B	108.9
C1—C2—H2B	109.2	H29A—C29—H29B	107.8
C3—C2—H2B	109.2	C29—C30—C31	113.2 (3)
H2A—C2—H2B	107.9	C29—C30—H30A	108.9
C4—C3—C2	112.8 (3)	C31—C30—H30A	108.9
C4—C3—H3A	109.0	C29—C30—H30B	108.9
C2—C3—H3A	109.0	C31—C30—H30B	108.9
C4—C3—H3B	109.0	H30A—C30—H30B	107.7
C2—C3—H3B	109.0	C30—C31—C32	108.0 (3)
H3A—C3—H3B	107.8	C30—C31—C33	107.1 (3)
C3—C4—C5	107.8 (3)	C32—C31—C33	114.6 (3)
C3—C4—C6	107.3 (3)	C30—C31—C41	108.5 (3)
C5—C4—C6	115.0 (3)	C32—C31—C41	111.9 (3)
C3—C4—C14	108.2 (3)	C33—C31—C41	106.5 (3)
C5—C4—C14	112.3 (3)	C31—C32—H32A	109.5
C6—C4—C14	106.0 (3)	C31—C32—H32B	109.5
C4—C5—H5A	109.5	H32A—C32—H32B	109.5
C4—C5—H5B	109.5	C31—C32—H32C	109.5
H5A—C5—H5B	109.5	H32A—C32—H32C	109.5
C4—C5—H5C	109.5	H32B—C32—H32C	109.5
H5A—C5—H5C	109.5	C37—C33—C34	113.4 (3)
H5B—C5—H5C	109.5	C37—C33—C31	111.6 (3)
C10—C6—C7	113.5 (3)	C34—C33—C31	116.8 (3)
C10—C6—C4	111.0 (3)	C37—C33—H33	104.5
C7—C6—C4	116.7 (3)	C34—C33—H33	104.5
C10—C6—H6	104.7	C31—C33—H33	104.5
C7—C6—H6	104.7	C36—C34—C35	106.2 (3)
C4—C6—H6	104.7	C36—C34—C28	108.5 (3)
C8—C7—C9	106.7 (3)	C35—C34—C28	108.7 (3)
C8—C7—C1	109.5 (3)	C36—C34—C33	109.7 (3)
C9—C7—C1	107.8 (3)	C35—C34—C33	115.1 (3)
C8—C7—C6	113.8 (3)	C28—C34—C33	108.5 (3)
C9—C7—C6	110.0 (3)	C34—C35—H35A	109.5
C1—C7—C6	108.9 (3)	C34—C35—H35B	109.5
C7—C8—H8A	109.5	H35A—C35—H35B	109.5
C7—C8—H8B	109.5	C34—C35—H35C	109.5
H8A—C8—H8B	109.5	H35A—C35—H35C	109.5
C7—C8—H8C	109.5	H35B—C35—H35C	109.5
H8A—C8—H8C	109.5	C34—C36—H36A	109.5
H8B—C8—H8C	109.5	C34—C36—H36B	109.5
C7—C9—H9A	109.5	H36A—C36—H36B	109.5
C7—C9—H9B	109.5	C34—C36—H36C	109.5
H9A—C9—H9B	109.5	H36A—C36—H36C	109.5
C7—C9—H9C	109.5	H36B—C36—H36C	109.5
H9A—C9—H9C	109.5	C38—C37—C33	110.5 (3)
H9B—C9—H9C	109.5	C38—C37—H37A	109.6
C6—C10—C11	110.1 (3)	C33—C37—H37A	109.6
C6—C10—H10A	109.6	C38—C37—H37B	109.6
C11—C10—H10A	109.6	C33—C37—H37B	109.6
C6—C10—H10B	109.6	H37A—C37—H37B	108.1
C11—C10—H10B	109.6	C39—C38—C37	113.6 (3)
H10A—C10—H10B	108.2	C39—C38—H38A	108.9
C10—C11—C12	113.6 (3)	C37—C38—H38A	108.9
C10—C11—H11A	108.9	C39—C38—H38B	108.9
C12—C11—H11A	108.9	C37—C38—H38B	108.9
C10—C11—H11B	108.9	H38A—C38—H38B	107.7
C12—C11—H11B	108.9	C38—C39—C40	107.8 (3)
H11A—C11—H11B	107.7	C38—C39—C41	109.1 (3)
C11—C12—C13	107.5 (3)	C40—C39—C41	111.9 (3)
C11—C12—C14	108.6 (3)	C38—C39—C45	110.8 (3)
C13—C12—C14	112.7 (3)	C40—C39—C45	110.2 (3)
C11—C12—C18	110.3 (3)	C41—C39—C45	107.1 (3)
C13—C12—C18	109.8 (3)	C39—C40—H40A	109.5
C14—C12—C18	108.0 (3)	C39—C40—H40B	109.5
C12—C13—H13A	109.5	H40A—C40—H40B	109.5
C12—C13—H13B	109.5	C39—C40—H40C	109.5
H13A—C13—H13B	109.5	H40A—C40—H40C	109.5
C12—C13—H13C	109.5	H40B—C40—H40C	109.5
H13A—C13—H13C	109.5	C42—C41—C39	111.6 (3)
H13B—C13—H13C	109.5	C42—C41—C31	114.8 (3)
C15—C14—C12	111.8 (3)	C39—C41—C31	115.9 (3)
C15—C14—C4	114.2 (3)	C42—C41—H41	104.3
C12—C14—C4	115.6 (3)	C39—C41—H41	104.3
C15—C14—H14	104.6	C31—C41—H41	104.3
C12—C14—H14	104.6	C43—C42—C41	113.2 (3)
C4—C14—H14	104.6	C43—C42—H42A	108.9
C16—C15—C14	112.3 (3)	C41—C42—H42A	108.9
C16—C15—H15A	109.1	C43—C42—H42B	108.9
C14—C15—H15A	109.1	C41—C42—H42B	108.9
C16—C15—H15B	109.1	H42A—C42—H42B	107.7
C14—C15—H15B	109.1	C44—C43—C42	109.3 (3)
H15A—C15—H15B	107.9	C44—C43—H43A	109.8
C17—C16—C15	110.0 (3)	C42—C43—H43A	109.8
C17—C16—H16A	109.7	C44—C43—H43B	109.8
C15—C16—H16A	109.7	C42—C43—H43B	109.8
C17—C16—H16B	109.7	H43A—C43—H43B	108.3
C15—C16—H16B	109.7	C43—C44—C45	110.7 (3)
H16A—C16—H16B	108.2	C43—C44—C49	120.1 (3)
C16—C17—C18	111.5 (3)	C45—C44—C49	105.3 (3)
C16—C17—C22	119.3 (3)	C43—C44—H44	106.6
C18—C17—C22	105.1 (3)	C45—C44—H44	106.6
C16—C17—H17	106.7	C49—C44—H44	106.6
C18—C17—H17	106.7	C47—C45—C44	100.1 (3)
C22—C17—H17	106.7	C47—C45—C46	106.2 (3)
C20—C18—C19	106.4 (3)	C44—C45—C46	110.7 (3)
C20—C18—C17	100.1 (3)	C47—C45—C39	117.5 (3)
C19—C18—C17	110.5 (3)	C44—C45—C39	110.1 (3)
C20—C18—C12	117.7 (3)	C46—C45—C39	111.6 (3)
C19—C18—C12	111.9 (3)	C45—C46—H46A	109.5
C17—C18—C12	109.5 (3)	C45—C46—H46B	109.5
C18—C19—H19A	109.5	H46A—C46—H46B	109.5
C18—C19—H19B	109.5	C45—C46—H46C	109.5
H19A—C19—H19B	109.5	H46A—C46—H46C	109.5
C18—C19—H19C	109.5	H46B—C46—H46C	109.5
H19A—C19—H19C	109.5	C45—C47—C48	103.0 (3)
H19B—C19—H19C	109.5	C45—C47—H47A	111.2
C21—C20—C18	104.1 (3)	C48—C47—H47A	111.2
C21—C20—H20A	110.9	C45—C47—H47B	111.2
C18—C20—H20A	110.9	C48—C47—H47B	111.2
C21—C20—H20B	110.9	H47A—C47—H47B	109.1
C18—C20—H20B	110.9	C47—C48—C49	105.3 (3)
H20A—C20—H20B	108.9	C47—C48—H48A	110.7
C20—C21—C22	106.3 (3)	C49—C48—H48A	110.7
C20—C21—H21A	110.5	C47—C48—H48B	110.7
C22—C21—H21A	110.5	C49—C48—H48B	110.7
C20—C21—H21B	110.5	H48A—C48—H48B	108.8
C22—C21—H21B	110.5	C50—C49—C44	115.2 (3)
H21A—C21—H21B	108.7	C50—C49—C48	112.6 (3)
C23—C22—C17	115.4 (3)	C44—C49—C48	104.3 (3)
C23—C22—C21	112.3 (3)	C50—C49—H49	108.1
C17—C22—C21	103.7 (3)	C44—C49—H49	108.1
C23—C22—H22	108.4	C48—C49—H49	108.1
C17—C22—H22	108.4	O6—C50—C51	106.0 (3)
C21—C22—H22	108.4	O6—C50—C49	106.5 (3)
O3—C23—C24	106.5 (3)	C51—C50—C49	114.7 (3)
O3—C23—C25	102.5 (3)	O6—C50—C52	103.3 (3)
C24—C23—C25	112.4 (3)	C51—C50—C52	111.9 (3)
O3—C23—C22	106.0 (3)	C49—C50—C52	113.3 (3)
C24—C23—C22	114.5 (3)	C50—C51—H51A	109.5
C25—C23—C22	113.7 (3)	C50—C51—H51B	109.5
C23—C24—H24A	109.5	H51A—C51—H51B	109.5
C23—C24—H24B	109.5	C50—C51—H51C	109.5
H24A—C24—H24B	109.5	H51A—C51—H51C	109.5
C23—C24—H24C	109.5	H51B—C51—H51C	109.5
H24A—C24—H24C	109.5	C53—C52—C50	103.7 (3)
H24B—C24—H24C	109.5	C53—C52—H52A	111.0
C26—C25—C23	104.0 (3)	C50—C52—H52A	111.0
C26—C25—H25A	111.0	C53—C52—H52B	111.0
C23—C25—H25A	111.0	C50—C52—H52B	111.0
C26—C25—H25B	111.0	H52A—C52—H52B	109.0
C23—C25—H25B	111.0	C54—C53—C52	103.8 (3)
H25A—C25—H25B	109.0	C54—C53—H53A	111.0
C27—C26—C25	103.0 (3)	C52—C53—H53A	111.0
C27—C26—H26A	111.2	C54—C53—H53B	111.0
C25—C26—H26A	111.2	C52—C53—H53B	111.0
C27—C26—H26B	111.2	H53A—C53—H53B	109.0
C25—C26—H26B	111.2	O5—C54—O6	121.0 (3)
H26A—C26—H26B	109.1	O5—C54—C53	128.2 (4)
O2—C27—O3	121.3 (4)	O6—C54—C53	110.8 (3)
			
O1—C1—C2—C3	−67.4 (4)	O4—C28—C29—C30	−69.8 (4)
C7—C1—C2—C3	55.4 (4)	C34—C28—C29—C30	53.9 (4)
C1—C2—C3—C4	−57.9 (4)	C28—C29—C30—C31	−55.4 (4)
C2—C3—C4—C5	−70.1 (4)	C29—C30—C31—C32	−71.5 (4)
C2—C3—C4—C6	54.3 (4)	C29—C30—C31—C33	52.4 (4)
C2—C3—C4—C14	168.3 (3)	C29—C30—C31—C41	167.0 (3)
C3—C4—C6—C10	174.8 (3)	C30—C31—C33—C37	173.2 (3)
C5—C4—C6—C10	−65.4 (4)	C32—C31—C33—C37	−67.0 (4)
C14—C4—C6—C10	59.3 (4)	C41—C31—C33—C37	57.3 (4)
C3—C4—C6—C7	−53.0 (4)	C30—C31—C33—C34	−54.0 (4)
C5—C4—C6—C7	66.8 (4)	C32—C31—C33—C34	65.7 (4)
C14—C4—C6—C7	−168.4 (3)	C41—C31—C33—C34	−170.0 (3)
O1—C1—C7—C8	−164.7 (3)	O4—C28—C34—C36	−49.2 (4)
C2—C1—C7—C8	74.5 (4)	C29—C28—C34—C36	−170.2 (3)
O1—C1—C7—C9	−49.0 (4)	O4—C28—C34—C35	−164.3 (3)
C2—C1—C7—C9	−169.8 (3)	C29—C28—C34—C35	74.7 (4)
O1—C1—C7—C6	70.3 (4)	O4—C28—C34—C33	69.9 (4)
C2—C1—C7—C6	−50.5 (4)	C29—C28—C34—C33	−51.1 (4)
C10—C6—C7—C8	59.9 (4)	C37—C33—C34—C36	−56.2 (4)
C4—C6—C7—C8	−71.2 (4)	C31—C33—C34—C36	171.9 (3)
C10—C6—C7—C9	−59.8 (4)	C37—C33—C34—C35	63.5 (5)
C4—C6—C7—C9	169.1 (3)	C31—C33—C34—C35	−68.4 (4)
C10—C6—C7—C1	−177.7 (3)	C37—C33—C34—C28	−174.5 (3)
C4—C6—C7—C1	51.2 (4)	C31—C33—C34—C28	53.6 (4)
C7—C6—C10—C11	164.0 (3)	C34—C33—C37—C38	164.2 (3)
C4—C6—C10—C11	−62.2 (4)	C31—C33—C37—C38	−61.4 (4)
C6—C10—C11—C12	58.0 (4)	C33—C37—C38—C39	58.1 (4)
C10—C11—C12—C13	70.9 (4)	C37—C38—C39—C40	70.5 (4)
C10—C11—C12—C14	−51.3 (4)	C37—C38—C39—C41	−51.2 (4)
C10—C11—C12—C18	−169.4 (3)	C37—C38—C39—C45	−168.8 (3)
C11—C12—C14—C15	−175.2 (3)	C38—C39—C41—C42	−175.3 (3)
C13—C12—C14—C15	65.8 (4)	C40—C39—C41—C42	65.5 (4)
C18—C12—C14—C15	−55.6 (4)	C45—C39—C41—C42	−55.4 (4)
C11—C12—C14—C4	51.9 (4)	C38—C39—C41—C31	50.7 (4)
C13—C12—C14—C4	−67.0 (4)	C40—C39—C41—C31	−68.5 (4)
C18—C12—C14—C4	171.6 (3)	C45—C39—C41—C31	170.7 (3)
C3—C4—C14—C15	57.8 (4)	C30—C31—C41—C42	59.1 (4)
C5—C4—C14—C15	−61.0 (4)	C32—C31—C41—C42	−60.0 (4)
C6—C4—C14—C15	172.6 (3)	C33—C31—C41—C42	174.0 (3)
C3—C4—C14—C12	−170.5 (3)	C30—C31—C41—C39	−168.4 (3)
C5—C4—C14—C12	70.7 (4)	C32—C31—C41—C39	72.6 (4)
C6—C4—C14—C12	−55.7 (4)	C33—C31—C41—C39	−53.4 (4)
C12—C14—C15—C16	54.6 (4)	C39—C41—C42—C43	55.0 (4)
C4—C14—C15—C16	−171.9 (3)	C31—C41—C42—C43	−170.5 (3)
C14—C15—C16—C17	−54.7 (4)	C41—C42—C43—C44	−54.9 (4)
C15—C16—C17—C18	58.4 (4)	C42—C43—C44—C45	58.5 (4)
C15—C16—C17—C22	−178.7 (3)	C42—C43—C44—C49	−178.4 (3)
C16—C17—C18—C20	174.1 (3)	C43—C44—C45—C47	172.8 (3)
C22—C17—C18—C20	43.4 (3)	C49—C44—C45—C47	41.5 (3)
C16—C17—C18—C19	62.1 (4)	C43—C44—C45—C46	61.0 (4)
C22—C17—C18—C19	−68.5 (4)	C49—C44—C45—C46	−70.3 (4)
C16—C17—C18—C12	−61.6 (4)	C43—C44—C45—C39	−62.9 (4)
C22—C17—C18—C12	167.7 (3)	C49—C44—C45—C39	165.8 (3)
C11—C12—C18—C20	−69.8 (4)	C38—C39—C45—C47	−68.2 (4)
C13—C12—C18—C20	48.5 (4)	C40—C39—C45—C47	51.1 (4)
C14—C12—C18—C20	171.7 (3)	C41—C39—C45—C47	173.0 (3)
C11—C12—C18—C19	54.0 (4)	C38—C39—C45—C44	178.2 (3)
C13—C12—C18—C19	172.2 (3)	C40—C39—C45—C44	−62.5 (4)
C14—C12—C18—C19	−64.6 (4)	C41—C39—C45—C44	59.4 (4)
C11—C12—C18—C17	176.9 (3)	C38—C39—C45—C46	54.8 (4)
C13—C12—C18—C17	−64.9 (4)	C40—C39—C45—C46	174.1 (3)
C14—C12—C18—C17	58.4 (4)	C41—C39—C45—C46	−64.0 (4)
C19—C18—C20—C21	71.8 (4)	C44—C45—C47—C48	−46.3 (4)
C17—C18—C20—C21	−43.3 (4)	C46—C45—C47—C48	68.9 (4)
C12—C18—C20—C21	−161.8 (3)	C39—C45—C47—C48	−165.4 (3)
C18—C20—C21—C22	27.6 (4)	C45—C47—C48—C49	34.1 (4)
C16—C17—C22—C23	84.2 (4)	C43—C44—C49—C50	89.6 (4)
C18—C17—C22—C23	−149.9 (3)	C45—C44—C49—C50	−144.8 (3)
C16—C17—C22—C21	−152.6 (3)	C43—C44—C49—C48	−146.5 (3)
C18—C17—C22—C21	−26.7 (4)	C45—C44—C49—C48	−20.8 (4)
C20—C21—C22—C23	124.6 (3)	C47—C48—C49—C50	117.4 (3)
C20—C21—C22—C17	−0.6 (4)	C47—C48—C49—C44	−8.2 (4)
C27—O3—C23—C24	−140.5 (3)	C54—O6—C50—C51	−98.6 (4)
C27—O3—C23—C25	−22.2 (4)	C54—O6—C50—C49	138.8 (3)
C27—O3—C23—C22	97.2 (3)	C54—O6—C50—C52	19.2 (4)
C17—C22—C23—O3	168.6 (3)	C44—C49—C50—O6	69.1 (4)
C21—C22—C23—O3	50.1 (4)	C48—C49—C50—O6	−50.3 (4)
C17—C22—C23—C24	51.5 (4)	C44—C49—C50—C51	−47.8 (5)
C21—C22—C23—C24	−67.0 (4)	C48—C49—C50—C51	−167.3 (3)
C17—C22—C23—C25	−79.6 (4)	C44—C49—C50—C52	−178.0 (3)
C21—C22—C23—C25	161.9 (3)	C48—C49—C50—C52	62.6 (4)
O3—C23—C25—C26	29.3 (4)	O6—C50—C52—C53	−26.6 (4)
C24—C23—C25—C26	143.3 (3)	C51—C50—C52—C53	87.0 (4)
C22—C23—C25—C26	−84.6 (4)	C49—C50—C52—C53	−141.5 (3)
C23—C25—C26—C27	−26.3 (4)	C50—C52—C53—C54	24.8 (4)
C23—O3—C27—O2	−173.8 (3)	C50—O6—C54—O5	176.5 (4)
C23—O3—C27—C26	5.6 (4)	C50—O6—C54—C53	−3.4 (4)
C25—C26—C27—O2	−167.0 (4)	C52—C53—C54—O5	166.0 (4)
C25—C26—C27—O3	13.6 (4)	C52—C53—C54—O6	−14.1 (5)

### Hydrogen-bond geometry (Å, °)

**Table:**

*D*—H···*A*	*D*—H	H···*A*	*D*···*A*	*D*—H···*A*
O1—H1···O4^i^	0.84	2.02	2.836 (4)	163
O4—H4···O2	0.84	2.03	2.858 (4)	169

Symmetry codes: (i) *x*−1/2, −*y*+3/2, −*z*+1.
